# “Floating” Stent in a Coronary Aneurysm Presenting as ST-Elevation Myocardial Infarction

**DOI:** 10.3390/jcdd10020059

**Published:** 2023-02-01

**Authors:** Daniel De Castro, Sergio García-Gómez, Fernando Domínguez, Carlos Arellano, Juan Francisco Oteo

**Affiliations:** Department of Cardiology, University Hospital Puerta de Hierro Majadahonda, 28220 Madrid, Spain

**Keywords:** ST-segment-elevation myocardial infarction, coronary aneurysm, coronary angiography, drug-eluting stent, computed tomography angiography

## Abstract

Coronary artery aneurysm (CAA) presenting as an ST-elevation myocardial infarction (STEMI) represents a clinical challenge due to the technical difficulties in the percutaneous management of this specific situation. Appropriate treatment for CAA depends on the precise clinical situation and consists of medical management, surgical resection, or/and stent placement. The high rate of complications during percutaneous intervention (distal thrombus embolization, no-reflow phenomenon, stent malposition, or dissection) makes emergent surgery a frequent situation in these cases. We present the case of a 50-year-old man with a STEMI due to thrombotic occlusion of CAA. Specific angiographic techniques and intracoronary imaging help with the percutaneous management of acute thrombotic occlusions in CAA, providing a less invasive approach than emergent surgery.

## 1. Introduction

Coronary artery aneurysm (CAA) is defined by a focal enlargement of the coronary artery exceeding the 1.5-fold diameter of the adjacent normal segment. CAA of the left main stem is rare and occurs in only 0.1% of the population [1]. CAA presenting as ST-elevation myocardial infarction (STEMI) might be a clinical challenge given the technical difficulties in percutaneous management [2]. Herein, we report a case in which specific angiographic techniques provide optimal management of CAA thrombotic occlusion.

## 2. Detailed Case Description 

A 50-year-old man was admitted to the emergency room due to chest pain. He had never previously displayed symptoms compatible with angina. The chest pain started one hour before hospital admission. The patient had a history of an idiopathic pulmonary embolism one year prior. He was under treatment with acenocoumarol. He accidentally suspended acenocoumarol one week prior. He had a familiar history of venous thromboembolic disease. His sister was diagnosed with deep vein thrombosis and his mother was diagnosed with an idiopathic pulmonary embolism in the past. Congenital thrombophilia disorders were never ruled out. The patient denied either tobacco or other toxic consumption. His vitals revealed a temperature of 36.3 °C, a heart rate of 98 beats per minute, blood pressure of 110/58 mmHg, and oxygen saturation of 97% on room air. A physical examination did not reveal either cardiac murmurs or signs of congestive heart failure. The ECG showed an anterior ST-segment-elevation myocardial infarction. Transthoracic echocardiogram showed a moderate reduction in left-ventricular ejection-fraction apical akinesia, and no valvular disease. A chest-X-ray showed no relevant findings.

Initial laboratory reports revealed leukocytosis and an initial elevated high-sensitivity troponin I at 321 ng/L (reference range level, 0.0–72.0 ng/L). It took 24 h for troponins to the peak. The peak troponin level was 264,010 ng/L. COVID-19 infection was ruled out with a PCR test and a serology test. Lipid panels evidenced lipid levels that were not well controlled. Total cholesterol level was 201 mg/dL (reference range level, 150.0–200.0 mg/dL). Low-density lipoprotein (LDL) level was 143 mg/dL (reference range level, 70.0–160.0 mg/dL). High-density lipoprotein (LDL) level was 47 mg/dL (reference range level, 35.0–75.0 mg/dL). Glycohemoglobin and glucose blood levels were normal. The patient denied bad control of ambulatory blood pressure.

**Figure 1:**
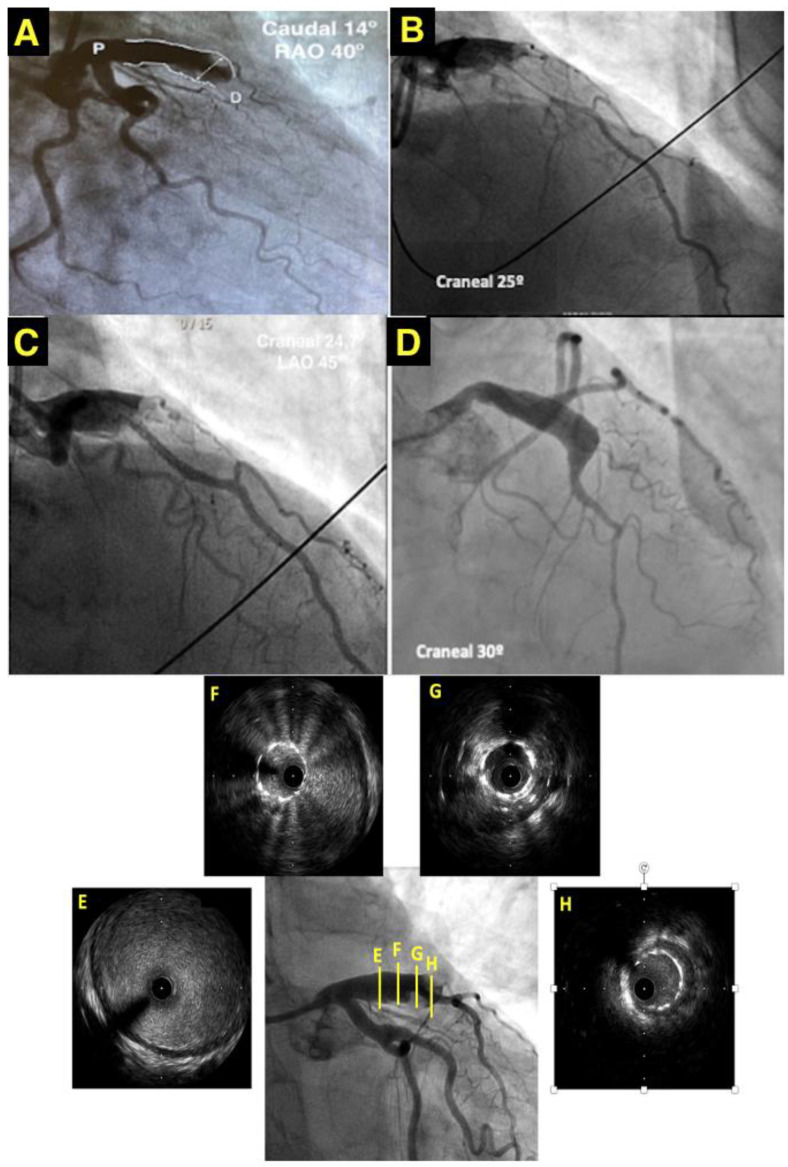
(**A**) CY showing a CAA with a maximum diameter of 8.49 mm (white double arrow). (**B**) CY displaying simultaneous contrast infusion from the microcatheter and support catheter. (**C**) CY showing aneurysm after stent implantation. (**D**) CY displaying the “funnel” shape of the distal segment of the aneurysm after the post-dilatation. (**E**–**H**) Coronary IVUS showing the stent apposition along the CAA in the ADA showing the stent “floating” in the aneurysm surrounded by thrombus. RAO: Right anterior oblique. LAO: Left anterior oblique.

The patient underwent an emergent coronary angiography due to this anterior ST-segment-elevation myocardial infarction. The coronary angiography revealed CAA, extending from the left main stem to the proximal segment of the anterior descendent artery (LAD) and a thrombotic occlusion (TIMI 1 flow) of its distal segment (Figure 1A/Video S1). Two guidewires were crossed to the distal segment of the LAD and the first diagonal. After percutaneous thrombus aspiration, the flow of the LAD was not restored. A perfusion of a glycoprotein IIb/IIIa inhibitor class (eptifibatide) was initiated during the procedure. A microcatheter was then crossed to the distal segment of the LAD and contrast was infused from the microcatheter and support catheter simultaneously in order to check the distal vessel’s diameter and to confirm the extension of the occlusion (Figure 1B/Video S1). Then, a 2.75 × 28 mm drug-eluted stent was implanted covering the occlusion, with its size adjusted to the distal segment of the aneurysm (Figure 1C). 

An intravascular ultrasound (IVUS) was performed thereafter, showing the stent “floating” in the aneurysm covered by thrombus (Figure 1E–H). A post-dilatation with a 3 non-compliant balloon in the distal segment of the stent improved the distal stent apposition and a post-dilatation with a 5 mm non-compliant balloon in the proximal segment resulted in a “funnel”-like shape (Figure 1D/Video S2). The LAD regained normal coronary flow (TIMI III). 

The patient was asymptomatic after the procedure. He was discharged from the hospital without any other complications. The patient resumed anticoagulant therapy with apixaban (5 mg) twice a day, as well as clopidogrel during the first year and aspirin during the first week. Angiotensin-converting enzyme inhibitors (rampiril, 2.5 mg once a day) and a beta-blocker (2.5 mg of bisoprolol once a day) were initiated due to persistent left-ventricular dysfunction after the procedure.

The patient was followed up in the outpatient clinic without incidence. The control echocardiogram showed a recovered left-ventricular ejection fraction in the third month after hospital discharge. A stress cardiac test was performed six months later. It did not show any signs of ischemia.

**Figure 2:**
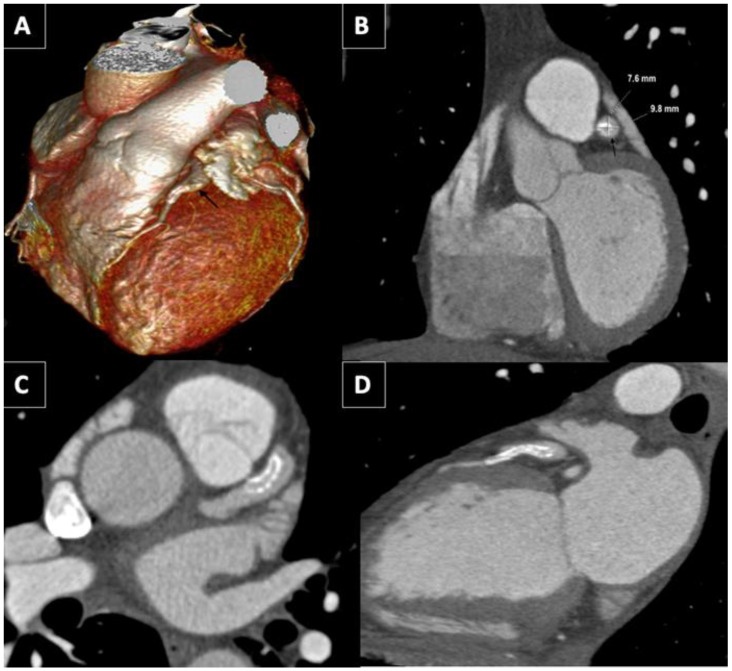
CCT. (**A**) GCA (black arrow) lateral view on three-dimensional volume-rendered CCT. (**B**) Coronal view of GCA (black arrow) with dimensions in CCT. Axial (**C**) and sagittal oblique (**D**) view of GCA showing the stent “floating” in the aneurysm with a “funnel”-like form disposition.

A coronary computed tomography angiography (CCTA) was carried out one year later to check the permeability of the stent. The CCTA showed a 7.6 × 9.8 mm diameter aneurysm (Figure 2A,B). The stent was “floating” in the aneurysm (Figure 2C,D) with a preserved distal flow. Acquired and congenital thrombophilia disorders were carried out. Clopidogrel was discontinued and patient agreed to be anticoagulated endlessly. The patient did not have angina two years after STEMI.

## 3. Discussion

We present a challenging and unusual case of CAA presenting as STEMI. CAA is defined by a focal enlargement of the coronary artery exceeding the 1.5-fold diameter of the adjacent normal segment. It is an uncommon condition that occurs in about 0.3–4.9% of patients undergoing coronary angiography. The most frequent location is the right coronary artery (40–70%). The left main stem location is particularly rare and occurs in only 0.1% of the population [1]. 

The pathophysiological mechanisms of CAA are not well understood, but atherosclerosis is the main identified etiology in adults [3]. Atherosclerosis and CAA commonly coexist, but only a limited subset of patients who present atherosclerosis develop CAAs, so genetic predisposition has been proposed as a hypothesis. Matrix metalloproteinases might be involved, because these enzymes play a role in the proteolysis of connective tissue proteins and a specific allele of a metalloproteinase gene (MMP3-5A) was found to be significantly more prevalent in patients with CAAs [1].

In relation to the atherosclerosis process, it has been noted that about one-third of CAAs is associated with obstructive coronary artery disease, so clinical presentation as an acute coronary syndrome is not rare and determines its management [4]. Apart from atherosclerosis, CAA may promote thrombosis through abnormal slow static flow and subsequent potential distal embolization [4], which could also contribute to myocardial ischemia. As atherosclerosis ought to be the main etiology of CAAs, patients should be treated determinedly with cholesterol-lowering therapies, including high-dose statin or PCSK9 inhibitors if necessary. Additionally, polymorphism of the angiotensin I enzyme may be a risk factor for developing CAAs, and the use of angiotensin-converting enzyme inhibitors should be studied in order to prevent CAA progression. Nevertheless, this remains to be proven in long-term studies [5]. 

Besides atherosclerosis, there are pathologies that could be associated with CAAs, including the following: congenital CAAs; conjunctive tissue diseases (Marfan syndrome, Ehlers–Danlos syndrome, fibromuscular dysplasia, neurofibromatosis); Kawasaki disease; Takayasu arteritis; infections (human immunodeficiency virus, bacterial, mycobacterial, syphilis, Lyme disease, mycotic aneurysm); drugs (cocaine, amphetamine, protease inhibitors); chest trauma; and tumors [6]. Percutaneous coronary intervention has also been reported as a trigger for aneurysm formation. The risk factors that have been reported for CAA development after drug-eluting stent implantation are lesion length > 33 mm, chronic total occlusion, lesion on the left anterior descending artery, and implantation in an infarct-related artery [7]. 

The “gold standard” for the diagnosis and evaluation of CAA is coronary angiography. Coronary angiography yields an evaluation of the entire coronary artery structure (anatomical variations, stenosis, collateral arteries, etc.). Likewise, it provides information on the aneurysm’s anatomy (shape, size, number, and location) and associated coronary artery disease, such as atherosclerosis [6]. Coronary angiography could underestimate the aneurysm size in case of intraluminal thrombi, as angiography consists of a ‘‘luminogram’’ and cannot detect a wall artery anomaly. Indeed, contrast stagnation and delayed antegrade contrast filling in the dilated coronary segment can hinder optimal coronary angiography imaging [6]. As shown in the case report, the use of a microcatheter. could be useful in solving this situation. The simultaneous infusion of contrast from the microcatheter and support catheter may yield an understanding of the extension of the thrombotic occlusion and the diameter of the vessel distal to it. 

A CCTA may be performed to complete the study of coronary structures. Moreover, it adds information about aneurysm structure and morphology. CCTA helps to capture complex anatomy and detect intraluminal thrombi. CCTA could assess the presence of associated coronary artery disease, as coronary angiography does [8]. Therefore, CCTCA may be a valuable strategy, for both diagnoses and follow-up, because it is non-invasive and rapid. Additionally, it provides a high anatomical definition. Three-dimensional reconstruction facilitates the study of CAA and the understanding of the relationship between the aneurysm and surrounding organs [8].

Other non-invasive imaging techniques, such as transthoracic echocardiography, have been shown to be of value in the investigation of CAAs, especially in the pediatric population [9]. The easy access and non-irradiation nature of this test explain why it is considered the first line of investigation in children with Kawasaki disease to rule out secondary CAAs. Indeed, it has both high sensitivity and high specificity in the evaluation of the proximal segment of the left main coronary artery and the right coronary artery in this disease [9]. Magnetic resonance imaging is also a potential imaging technique for the diagnoses and evaluation of CAAs. It could be a non-ionizing alternative to CCTA [8].

Regarding the management of CAA, there is a substantial knowledge gap due to the lack of randomized trials or large-scale data, so most of the current recommendations are based on small case series or anecdotal evidence. The appropriate treatment for CAA depends on the precise clinical situation and consists of medical management, surgical resection, or/and stent placement. Some algorithms have been published [10], which include the use of intracoronary imaging, specifically IVUS. The intracoronary images of the coronary arteries provide information on the composition of the lumen and the arterial wall [11]. The application of these techniques is probably one of the greatest advances in this field in recent years. The benefit of these tools is evident, as is seen in the case previously presented.

Some studies have shown that the percutaneous coronary intervention PCI has emerged as a long-term safe and effective strategy compared to coronary artery bypass grafting (CABG), and the use of drug-eluting stents appears to be associated with better outcomes [12,13]. It is important to highlight that most published cases cover symptomatic patients. Consequently, there is a lack of data for asymptomatic CAAs.

Concerning our case, management of CAA during acute coronary syndrome represents a unique clinical challenge, particularly in the context of profound thrombus burden [2]. Most cases need surgical intervention or percutaneous coronary intervention, and percutaneous techniques in this field have an increased risk of complications, such as distal thrombus embolization, no-reflow phenomenon, stent malposition, dissection, and rupture [6]. In order to succeed in cases with percutaneous management, several techniques were reported in the literature, such as covered-stent implantation, coil embolization, and stent-assisted coil insertion [14]. The “funnel”-like shape of the drug-eluting stent was performed to improve stent apposition and flow in the LAD.

The role of dual antiplatelet agents or therapeutic anticoagulation in the management of CAA is an area of ongoing debate. In the Coronary Artery Aneurysm Registry (CAAR) [13], antiplatelet therapy was found to be widely used. Aspirin was the most prescribed treatment (90.2%), with 64.8% of patients receiving dual antiplatelet therapy for a median duration of 12 months. Additional anticoagulants or prolonged dual antiplatelet therapy could be considered for aneurysms with multivessel disease or in cases of high thrombotic risk, and they were prescribed in only 13.4% of patients [13]. Recent studies have suggested a possible advantage of anticoagulation in patients with acute coronary syndrome [15]. A retrospective study suggested that more intense and prolonged antithrombotic treatment may result in lower mortality rates [12]. Nowadays, the use of direct anticoagulants is widespread, but experience in CAA is also scarce and based on case reports [15]. Glycoprotein IIb/IIIa receptor inhibitors may be an adjunctive therapy to PCI, due to the frequency of major thrombus burden, mainly in acute coronary syndrome [16].

## 4. Conclusions

To the best of our knowledge, this is a novel case due to the management performed from the point of view of antithrombotic treatment, the type of stent used, and follow-up through intracoronary imaging techniques. Tailored management with specific angiographic techniques is feasible in CAA presenting as STEMI, enabling an alternative and less invasive approach than emergent surgery in these patients.

## Data Availability

Please note that no data set was generated due to the nature of this publication.

## Supplementary Materials

The following supporting information can be downloaded at: https://www.mdpi.com/article/10.3390/jcdd10020059/s1, Video S1: Coronary angiography showing a coronary artery aneurysm, from left main stem to the proximal segment of anterior descendent artery. Simultaneous contrast infusion from the microcatheter and support catheter showing the extension of the thrombotic occlusion. Video S2: Coronary angiography showing the “funnel”-like form disposition of the stent after post-dilatation.
